# The Slovenian Version of the Thirst Distress Scale for Patients with Heart Failure: Translation and Psychometric Evaluation

**DOI:** 10.3390/healthcare12242564

**Published:** 2024-12-20

**Authors:** Špela Plesec, Andreja Kvas, Mitja Lainscak, Tiny Jaarsma, Nana Waldréus, Jerneja Farkas

**Affiliations:** 1 Faculty of Medicine, University of Ljubljana, 1000 Ljubljana, Slovenia; mitja.lainscak@guest.arnes.si (M.L.); jerneja.farkas@sb-ms.si (J.F.); 2 Faculty of Health Sciences, University of Ljubljana, 1000 Ljubljana, Slovenia; 3 Division of Cardiology, General Hospital Murska Sobota, 9000 Murska Sobota, Slovenia; 4 Department of Health, Medicine, and Caring Sciences, Linköping University, 581 83 Linköping, Sweden; 5 Department of Cardiology, University Medical Center Utrecht, 3584 CX Utrecht, The Netherlands; 6 Department of Neurobiology, Care Sciences and Society, Division of Nursing, Karolinska Institutet, 171 77 Huddinge, Sweden; 7 Department of Research, General Hospital Murska Sobota, 9000 Murska Sobota, Slovenia; 8 National Institute of Public Health, 1000 Ljubljana, Slovenia

**Keywords:** heart failure, thirst, psychometric, validity, reliability

## Abstract

Thirst is a common symptom in patients with heart failure (HF), which can cause a high degree of distress. Increased fluid intake due to thirst can lead to clinical deterioration because of shifts in volume status. The thirst assessment should be included in routine patient follow-up; however, only a limited set of validated instruments are available. Aims: To translate and evaluate psychometric properties of the Slovenian version of the Thirst Distress Scale for Patients with Heart Failure (TDS-HF). Methods: The English version of the TDS-HF was translated back and forth into the Slovenian language. The psychometric evaluation included content validity, internal consistency, construct validity, and concurrent validity assessments. For this purpose, we also evaluated thirst intensity using a visual analogue scale (VAS, 100 mm). Results: Overall, 159 patients with HF (mean age 71 ± 9 years, 69% male, 77% in the New York Heart Association (NYHA) functional class II-III, mean Left Ventricular Ejection Fraction (LVEF) 36 ± 12%) completed the TDS-HF. Confirmatory factor analysis (CFA) supported the one-factor structure of the scale. The Slovenian version demonstrated good content validity (scale content validity index based on the average method (S-CVI/Ave) 0.96) and internal consistency (Cronbach’s alpha 0.83). In terms of concurrent validity, a positive correlation was observed between TDS-HF and thirst intensity (r = 0.36, *p* ≤ 0.01). Conclusions: The psychometric evaluation of the Slovenian version of the TDS-HF showed adequate psychometric properties, indicating that the scale is suitable for clinical practice and research in Slovenia to measure thirst distress in patients with HF.

## 1. Introduction

Heart failure (HF) is a complex and life-threatening syndrome that impacts 1–3% of the general adult population [1]. Its prevalence is on the rise, driven by factors such as the growing aging population, advancements in the treatment and survival rates of ischemic heart disease, and the accessibility of evidence-based therapies that effectively extend the lives of individuals with HF [1,2,3]. Slovenian data replicate global epidemiology, with HF affecting 1 in 4 patients over 65 hospitalized in internal medicine departments, accounting for approximately 16,000 annual hospitalizations, and leading to 40% readmission and 20% mortality within a year of discharge [4].

Among other symptoms, thirst has been reported as an important cause of distress for patients with HF [5,6]. Thirst distress refers to the degree to which the patient is bothered by thirst or the discomfort associated with it [5]. In previous studies, it has been measured with a Thirst Distress Scale for Patients with HF (TDS-HF). TDS-HF was originally developed from a Thirst Distress Scale for Patients with Renal Failure [7], which was then simultaneously translated into Swedish and English language [8]; thereafter, German [9] and Turkish [10] translations followed. Measuring psychological distress and thirst distress separately is essential, as they have different causes and impacts, allowing for more targeted and effective care [11].

Previous studies have reported that a significant proportion of patients with HF, both in hospital and outpatient settings, experience moderate to severe thirst distress [12,13]. Furthermore, in patients who reported a TDS-HF score increase, this related to a decrease in their health-related quality of life [14]. Patients with high thirst intensity were found to have higher thirst distress [13], and a prospective 4-week follow-up study revealed that patients with elevated thirst distress upon admission continued to experience higher levels of thirst distress over time [15].

Thirst is a crucial component of the well-being of HF patients and is also linked to outcomes. Increased fluid intake driven by thirst can cause clinical deterioration due to changes in volume status. Diuretics may further exacerbate thirst by causing fluid loss and electrolyte imbalances, triggering the body’s natural urge to rehydrate [5].

At the time of the study, there was no valid tool available to assess thirst distress in Slovenian-speaking patients with HF. Therefore, the aim of this study was to translate the TDS-HF from English to Slovenian and to evaluate its psychometric properties. The research question of this study was “How reliable and valid is the Slovenian version of the TDS-HF?”

## 2. Methods

### 2.1. Study Design

This study was designed as a cross-sectional validation study to assess the psychometric properties of the TDS-HF in a Slovenian patient population with HF.

### 2.2. Study Sample

The study included patients with HF who were either receiving care at outpatient clinics or hospitalized in the cardiology department of a regional hospital in Slovenia due to HF deterioration.

### 2.3. Inclusion and Exclusion Criteria

The inclusion criteria were HF, age ≥ 18 years, and the ability to read and write in Slovenian. Patients with dementia or other mental disorders that could potentially impact their ability to respond to questionnaires were excluded.

### 2.4. Recruitment Procedure

Participants were recruited using a convenience sampling method. The researcher personally invited patients to take part in the study and complete the questionnaires either during their outpatient clinic visit or within 72 h of hospital admission. Data collection occurred between September 2021 and January 2023.

### 2.5. Measurements

#### 2.5.1. Thirst Distress Scale

The Slovenian version of TDS-HF was used to quantify patients’ experience with thirst over the past 2–3 days. TDS-HF is a 5-point Likert scale comprising eight statements relating to thirst. Patients were requested to rate these statements on a scale from 1 (strongly disagree) to 5 (strongly agree). The total TDS-HF score ranges from 8 to 40, with higher scores indicating greater thirst distress [8].

#### 2.5.2. Visual Analogue Scale

A visual analogue scale (VAS; 0–100 mm) was used to assess thirst intensity at the moment of questioning. Patients were asked to grade their thirst, ranging from no thirst on the left (0) to worst possible thirst on the right (100), by marking a cross on the line [16].

#### 2.5.3. Sociodemographic and Clinical Data

Sociodemographic data included age, gender, household composition (alone/with partner/with others), and educational level (primary school, secondary school, higher education). Clinical data included smoking status, Left Ventricular Ejection Fraction (LVEF), the New York Heart Association (NYHA) functional class (I-IV), cardiac and non-cardiac comorbidities, self-reported fluid and salt restriction, and pharmacological treatment. Data were collected during the interview or later from patients’ medical records.

### 2.6. Psychometric Properties

Construct validity of the TDS-HF was assessed through a one-dimensional confirmatory factor analysis (CFA), based on the original validation [8].

Concurrent validity was evaluated by examining the correlation between TDS-HF scores and thirst intensity, as assessed using VAS.

The TDS-HF was translated into Slovenian following established guidelines [17], with permission from the original authors [8]. The forward translation was independently completed by two native Slovenian experts, followed by a back translation into English by two native English speakers who were blinded to the original scale. After consulting with local experts (dietitians, nurses, and cardiologists), the final version was reviewed by the original authors, who suggested no further modifications. Pilot testing with HF patients confirmed clarity and cultural relevance.

Content validity for each item and the overall TDS-HF was assessed using a panel of nine independent experts (a dietitian, three nurses, three public health researchers, a psychologist, and a sociologist), who rated the relevance of each item on a 4-point scale (1—item is not relevant, 2—item is somewhat relevant, 3—item is quite relevant, and 4—item is highly relevant). The scale was sent to nine experts, all of whom provided feedback. Item content validity indices (I-CVI) were calculated by dividing the number of experts rating the item as either 3 or 4 by the total number of experts. The scale-level content validity index (S-CVI/Ave) was determined by averaging the I-CVIs across items. According to Lynn [18], an I-CVI of at least 0.78 with 6 to 10 experts and an S-CVI/Ave of 0.90 or above indicate good content validity.

### 2.7. Statistical Analysis

Descriptive variables are presented as frequencies (%), while numeric variables are presented as mean (standard deviation). The mean value and distribution of responses were calculated for each item of TDS-HF. Additionally, the mean value of thirst intensity (VAS, 0–100 mm) and the total TDS-HD score were also calculated.

To assess the construct validity of TDS-HF, we conducted a CFA. Before conducting the analysis, we assessed the appropriateness of the method by examining the Kaiser–Meyer–Olkin (KMO > 0.5) measure of sampling adequacy and conducting Bartlett’s test of sphericity (*p* < 0.05). CFA was conducted using the unweighted least squares estimation method due to the non-normal distribution of the data. A value of Goodness-of-Fit Index (GFI) ≥ 0.95 is considered an excellent fit [19]. A Normed-fit-index value (NFI) of 1 shows a perfect fit, while models valued at < 0.9 can usually be improved substantially [20]. Relative-fit-index (RFI) values close to 1 indicate a very good fit, while 1 indicates a perfect fit [21].

The measurement of internal consistency as a form of reliability testing was conducted using Cronbach’s α coefficient. According to Tavakol and Dennick [22], Cronbach’s alpha (α) should ideally range between 0.70 and 0.90. Spearman’s rank correlation coefficient was used to calculate inter-item correlations and item-total correlations. Item-total correlations should range between 0.30 and 0.70 and the inter-item correlation should be between 0.2 and 0.4 [23].

The concurrent validity of TDS-HF with thirst intensity measured with VAS (0–100 mm) was calculated using the Spearman rank correlations coefficient. Typically, concurrent validity scores fall within a range of 0 to 1, with interpretations as follows: less than 0.25 indicates small concurrence, 0.25 to 0.50 suggests moderate concurrence, 0.50 to 0.75 signifies good concurrence, and over 0.75 indicates excellent concurrence [24].

All statistical analyses, except CFA, were conducted using IBM SPSS version 26 (SPSS Inc., Armonk, NY, USA) at a significance level of *p* < 0.05 (α = 0.05). CFA was conducted using the add-in for IBM SPSS Amos version 26.

## 3. Results

### 3.1. Patients’ Characteristics

One hundred and fifty-nine patients with HF completed the TDS-HF and were included in the final analysis. Almost two-thirds of those enrolled were recruited from outpatient HF clinics (65%). The mean age was 71 (±9) years and most of them were males (69%). The majority of patients were cohabiting (75%) and had completed either a primary (46%) or secondary (44%) level of education. On average, patients’ LVEF was 36% (±12). Most of them were non-smokers (87%) and were classified as NYHA functional class II (34%) or III (43%). The most common non-cardiac comorbidities were chronic kidney disease (CKD) (40%), type 2 diabetes (33%), and malignancy (16%). The most prevalent cardiac comorbidities included arterial hypertension (68%), atrial fibrillation (56%), and ischemic heart disease (38%). They also reported that they have received advice on fluid (60%) and salt (45%) restrictions and most of them were receiving Renin–Angiotensin–Aldosterone System Inhibitors (RAASi) (86%), beta-blockers (87%), diuretics (75%), and mineralocorticoid receptor antagonists (MRA) (56%), respectively (Table 1).

### 3.2. Analysis of TDS-HF Items

**Figure 1:**
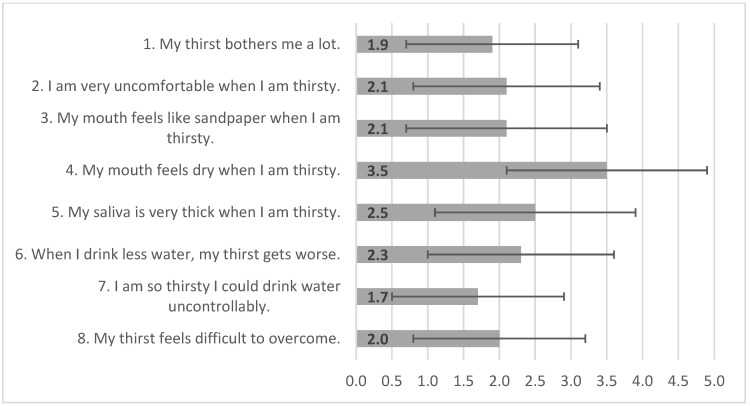
Mean and standard deviation (SD) scores of the items on the TDS-HF (n = 159).

**Figure 2:**
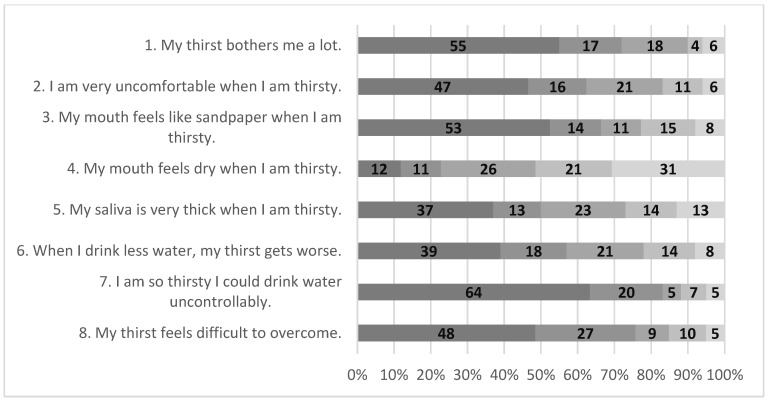
Item distribution of TDS-HF (n = 159).

All 159 patients participating in this study successfully completed all 8 items of the TDS-HF. Item analysis revealed that item 4, “My mouth feels dry when I am thirsty”, had the highest mean value of 3.5 ± 1.4, and item 7, “I am so thirsty I could drink water uncontrollably”, had the lowest mean value of 1.7 ± 1.2 (Figure 1). Almost half or more of the patients answered that they strongly disagree with statements of item 1, “My thirst bothers me a lot” (55%), item 3, “My mouth feels like sandpaper when I am thirsty” (53%), and item 7, “I am so thirsty I could drink water uncontrollably” (64%). For item 4, “My mouth feels dry when I am thirsty”, most patients (77%) responded with scores 3 to 5, which indicates moderate to strong agreement with this statement (Figure 2).

### 3.3. Construct Validity

CFA was conducted and the factor loadings of all items were found to range from 0.54 to 0.74 (Table 2). The model fit of the unidimensional TDS-HF was deemed acceptable, with a Goodness-of-Fit Index (GFI) of 0.99, a Normed-fit Index (NFI) of 0.97, and a Relative-fit Index (RFI) of 0.96. The correlation matrix values varied between 0.23 (items 7 and 5) and 0.59 (items 8 and 7) (Table 3).

### 3.4. Content Validity

The assessment of content validity involved I-CVI, which ranged between 0.89 and 1. Additionally, the S-CVI/Ave was calculated to be 0.96.

### 3.5. Internal Consistency Reliability

The internal consistency of the TDS-HF analyzed with Cronbach’s alpha (α) was 0.83. We also examined Cronbach’s alpha when deleting one item, which ranged from 0.79 to 0.81. If we exclude any TDS-HF item, Cronbach’s alpha decreases. The item-total correlations ranged from 0.62 to 0.75. The item with the highest correlation was item 8 (“My thirst feels difficult to overcome”), while the item with the lowest correlation was item 4 (“My mouth feels dry when I am thirsty”). Inter-item correlations ranged from 0.23 to 0.59. The strongest correlation was observed between item 8 (“My thirst feels difficult to overcome”) and item 7 (“I am so thirsty I could drink water uncontrollably”), while the weakest correlation was found between item 7 (“I am so thirsty I could drink water uncontrollably”) and item 5 (“My saliva is very thick when I am thirsty”) (Table 3).

### 3.6. Concurrent Validity

The mean value of thirst intensity (VAS, 0–100 mm) for all 159 patients in this study was 38.70 ± 24.57 and the mean value of the TDS-HF total score was 18.13 ± 6.93. Furthermore, a positive correlation was observed between thirst distress (TDS-HF) and thirst intensity (VAS), with a correlation coefficient of r = 0.36 (*p* ≤ 0.01).

## 4. Discussion

We performed the first study that translated and psychometrically evaluated the Slovenian version of TDS-HF. Methodologically, we followed the authors of the original version [8] and the subsequent German translation [9], with the primary aim of enabling direct comparisons between the results and verification of findings of previous research.

The Slovenian version of the TDS-HF demonstrated good content validity and internal consistency. CFA supported the one-factor structure of the scale. Reassuringly, a positive correlation was observed between TDS-HF and thirst intensity in terms of concurrent validity.

Regarding construct validity, we performed a CFA, which revealed a one-dimensional scale structure with acceptable values of calculated indices [19,20,21]. Based on our results, we can support the results from developers [8] and the authors of German [9] and Turkish [10] translations that TDS-HF measures a single component, which is thirst distress.

The concurrent validity of the TDS-HF was indicated by a positive correlation with thirst intensity measured with VAS, with a correlation coefficient of r = 0.36 (*p* ≤ 0.01). The correlation between these two measurements was moderate and compared to the original version, r = 0.56 (*p* < 0.001) [8], and the German translation, r = 0.72 (*p* ≤ 0.001) [9], not as high, but still statistically significant. We have confirmed the need for separate tools to measure different dimensions of thirst, such as intensity and distress, also in the Slovenian language.

Our Cronbach’s alpha coefficient was 0.83: although somewhat lower than for the original English (0.90) [8] and German version (0.89) [9], our result indicates a high level of internal consistency [22]. Also, item-total and inter-item correlations were mostly within the recommended values from Field [23].

In this study, the content validity was established by obtaining ratings from nine experts. Values for both I-CVI and S-CVI/Ave exceeded the CVI threshold of 0.78, as recommended by Lynn [18]. In other studies [8,9,10], the assessment of content validity by calculating CVI was not conducted, making it impossible to make comparisons.

Instruments aimed at assessing patient perception, experience, and performance are always challenged by culturally driven differences. This challenge was evident during the global expansion of the TDS-HF, highlighting the need to accommodate language and cultural specifics. As noted by the authors of the previous version of TDS-HF, there were some linguistic and cultural differences in the translation of one item on the scale. Item 3, “My mouth feels like sandpaper when I am thirsty”, was described in lay English as “cotton”, in the Japanese version as “rough”, in the German version as “furry” and in the Swedish and Dutch versions, the word “sandpaper” was used [8,9]. The term “sandpaper” was also the most appropriate expression to describe the feeling in the mouth when someone is thirsty in the Slovenian language. This item appears to be challenging in several language versions, and it may be worth considering its exclusion in a new version of the TDS-HF. However, in our sample, excluding this item did not alter the internal consistency of the TDS-HF.

The TDS-HF is a valuable tool in both research and clinical settings for systematically assessing thirst distress in HF patients, a factor that significantly impacts quality of life yet is often overlooked [5,6,12]. In research, the TDS-HF provides reliable, valid measurements [8,9,10] that facilitate cross-study comparisons, support the identification of factors influencing thirst distress, and contribute to a deeper understanding of the patient experience. Clinically, the scale helps identify patients with significant thirst distress, enabling personalized management strategies such as patient education on thirst management or adjustments to fluid intake guidelines, particularly for those under fluid restrictions. Additionally, monitoring changes in thirst distress is crucial for healthcare providers, especially in managing patients with HF [15]. Research indicates that fluctuations in thirst levels can signal alterations in a patient’s HF status or the effectiveness of interventions [25], thereby enhancing patient care and advancing research in HF management.

In our study, the mean value of thirst intensity (VAS, 0–100 mm) was 38.7 ± 24.6, with a median score of 47 (Interquartile Range (IQR) 20–52). Comparatively, other studies have reported median thirst intensity scores of 16 mm (IQR 4–44) [8] and 39 mm (IQR 22–58) [9], while the mean value of thirst distress (TDS-HF, 8–40) was 18.1 ± 6.9, with a median of 17 (IQR 12–23). These findings suggest that, according to the predefined categories established by Eng et al. [26], the patients with HF in Slovenia experienced moderate thirst distress on average. When comparing these findings to the results reported by the developers of the scale, which included patients from Sweden, the Netherlands, and Japan, their mean value of TDS-HF was similar to 16.4 ± 7.8 [8]. Also, results from Germany showed comparable values of mean 20 ± 8 and median 18 (IQR, 13–27) [9]. It is reasonable to compare the results of our research with those of countries that share similar climatic conditions, cultural backgrounds, and food-related habits, as these factors can potentially influence the sensation of thirst and associated distress. Considering this, the results of our study are highly comparable to those observed in Germany.

## 5. Limitations

Some limitations should be acknowledged. Participant recruitment relied on convenience sampling, which may limit the generalizability of the findings. Nonetheless, Slovenia is a compact country with minor ethnic, language, and religious variety; thus, results should be nationally applicable. Also, the data-gathering process was prolonged due to the limitations imposed by the COVID-19 pandemic.

## 6. Conclusions

The psychometric evaluation of the Slovenian version of the TDS-HF revealed strong construct, concurrent, and content validity. It also demonstrated high reliability in terms of internal consistency. The psychometric properties of the TDS-HF were comparable to those reported in previous studies and exhibited adequate characteristics, suggesting that the scale is suitable for clinical practice and research in Slovenia to measure thirst distress in patients with HF.

## Figures and Tables

**Table 1:** Characteristics of study sample (n = 159).

Sociodemographic Characteristics		
age (years) mean (SD), range		71 (±9), 45–90
gender, male n (%)		110 (69)
Household Composition n (%)	alone	39 (25)
	with partner	78 (49)
	with others	41 (26)
Education n (%)	primary school	72 (46)
	secondary school	70 (44)
	higher education	16 (10)
Setting n (%)	outpatient HF clinic	103 (65)
	hospital	56 (35)
Clinical characteristics		
smokers n (%)		20 (13)
LVEF mean (SD)		36 (±12)
NYHA n (%)	class I	6 (4)
	class II	54 (34)
	class III	69 (43)
	class IV	30 (19)
Comorbidities n (%)		
Diabetes type 2		52 (33)
Anemia		17 (11)
COPD		16 (10)
Stroke		12 (8)
CKD		64 (40)
Malignancy		26 (16)
Arterial hypertension		108 (68)
Atrial fibrillation		89 (56)
Ischemic heart disease		61 (38)
Non-pharmacological treatment n (%)		
Fluid restriction		95 (60)
Salt restriction		69 (45)
Pharmacological treatment n (%)		
Diuretics		119 (75)
ACE		71 (45)
ARB		10 (6)
ARNI		56 (35)
Beta-blocker		138 (87)
MRA		90 (57)
SGLT2 inhibitors		39 (25)
Omeprazol		70 (44)
Opioids		14 (9)
Antidepressants		19 (12)

Abbreviation: ACE, Angiotensin-converting Enzyme Inhibitor; ARB, Angiotensin Receptor Blocker; ARNI, Angiotensin Receptor Neprilysin Inhibitor; CKD, chronic kidney disease; COPD, chronic obstructive pulmonary disease; HF, heart failure; LVEF, Left Ventricular Ejection Fraction; MRA, Mineralocorticoid Receptor Antagonist; NYHA, the New York Heart Association.

**Table 2:** Confirmatory factor analysis: factor loadings of the TDS-HF.

TDS-HF	Factor Loadings
Item 1	0.55
Item 2	0.62
Item 3	0.69
Item 4	0.54
Item 5	0.56
Item 6	0.58
Item 7	0.62
Item 8	0.74

**Table 3:** Item-total correlations, inter-item correlations, and Cronbach’s alpha (α) of the TDS-HF (n = 159).

	Item-Total Correlations	Inter-Item Correlations							
Item	TDS-HF (n = 159)	1	2	3	4	5	6	7	8
1. My thirst bothers me a lot.	0.62	1							
2. I am very uncomfortable when I am thirsty.	0.68	0.42 **	1						
3. My mouth feels like sandpaper when I am thirsty.	0.73	0.27 **	0.46 **	1					
4. My mouth feels dry when I am thirsty.	0.63	0.27 **	0.36 **	0.33 **	1				
5. My saliva is very thick when I am thirsty.	0.65	0.35 **	0.30 **	0.44 **	0.40 **	1			
6. When I drink less water, my thirst gets worse.	0.65	0.26 **	0.29 **	0.45 **	0.36 **	0.35 **	1		
7. I am so thirsty I could drink water uncontrollably.	0.67	0.44 **	0.33 **	0.44 **	0.26 **	0.23 *	0.38 **	1	
8. My thirst feels difficult to overcome.	0.75	0.41 **	0.52 **	0.49 **	0.36 **	0.35 **	0.39 **	0.59 **	1
Average item-total correlation	0.55								
Cronbach α TDS-HF (8 items)	0.83								
Cronbach α when deleting one item of TDS-HF	0.79–0.81								

Abbreviation: TDS-HF, Thirst Distress Scale for Patients with Heart Failure. * p < 0.002, ** p < 0.001.

## Data Availability

The data presented in this study are available on request from the corresponding author due to ethical reasons.
